# HIV positivity and referral to treatment following index testing of partners and children of HIV-infected patients in public sector facilities in South Africa

**DOI:** 10.1097/QAI.0000000000002048

**Published:** 2019-08-01

**Authors:** Dvora Joseph Davey, Kristin Marie Wall, Claire Serrao, Marlien Prins, Madaline Feinberg, Ntokozo Mtonjana, Khanyo Hlophe, Lindiwe Zuma, Senate Sejake, Todd Malone

**Affiliations:** 1. Department of Epidemiology, Fielding School of Public Health, University of California, Los Angeles; 2. Division of Epidemiology and Biostatistics, School of Public Health and Family Medicine, University of Cape Town, South Africa; 3. School of Public Health, Emory University, Atlanta GA, USA; 4. BroadReach Healthcare, Cape Town, South Africa; 5. Department of Health, Alfred Nzo District, South Africa; 6. Department of Health, Harry Gwala District, South Africa; 7. Department of Health, Sedibeng District, South Africa

**Keywords:** Index testing, positivity, HIV testing and counseling, couples, partners, children

## Abstract

**Background:**

There is an imperative need for innovative interventions to identify people living with HIV and initiate them on antiretroviral therapy (ART). The objective of this study was to determine the feasibility of providing index partner/child testing of people living with HIV.

**Methods:**

We trained 86 nurses and counsellors in 56 public health facilities in six high HIV burden Districts in 2017 to provide index partner/child testing (tracing and testing of partners/children of people living with HIV). We collected programmatic data including index partner/child HIV positivity by age, gender and location of testing. In sub-analyses, we evaluated factors associated with identifying HIV-positive partners and children in separate models using multivariable logistic regression.

**Results:**

We tested 16,033 partners and children of index patients between October 2017 and June 2018. Most of those tested were female (61%) and 20–39 years old (39%). Overall, 6.4% were 10–14 years old, 9.5% were 15–19 years; 8% were >50 years. HIV positivity was 38% (95% CI=36%−40%). In children ages 10–14, 13% were HIV-infected (95% CI=11%−14%). In subanalyses, HIV positivity in partners was associated with their increased age (adjusted odds ratio [aOR] for increase in 5-year age category=1.21; 95% CI=1.04, 1.42), female gender (aOR=1.38; 95% CI=1.04, 1.82) and bringing the partner in for HIV testing vs. referring the partner through the provider or recommending testing to the partner (aOR=1.94, 95% CI=1.43, 2.63), adjusting for location of testing. Almost all patients diagnosed (97%) were referred to ART.

**Conclusion:**

Providing index partner/child testing was feasible and we identified a very high yield when testing partners/children of index patients. Index partner/child testing should be offered to all patients living with HIV to improve case finding.

## Background:

In South Africa, the country with the largest HIV epidemic and antiretroviral therapy (ART) program in the world, an estimated 85% of HIV-infected adults knew their status and 71% of those are on ART. Of those on ART who had a recent viral load test, 88% had achieved viral load suppression (viral suppression is considered less than 1000 copies per ml by the South African Department of Health) as of 2017 [1]. Innovative, cost-effective and scalable approaches such as partner notification and index partner testing strategies are needed to meet UNAIDS 90–90-90 goals, which refer to 90% of people living with HIV knowing their HIV status, 90% of those on ART and 90% of people on ART virally suppressed (VLS) [1]. A recent systematic review demonstrated the dearth of research on HIV partner notification strategies, with only ten studies identified [2]. The review highlighted the effectiveness of partner assisted referrals, mainly assisted by healthcare providers, compared to passive referral, not assisted by healthcare providers, in improving partner testing, notification of serostatus, yield of new positives, and linkage to ART.

Additionally, the same HIV testing and treatment cascade is poorer in men with only 78% of men living with HIV diagnosed, 67% of those on ART and 82% virally suppressed [1]. There is a current, imperative need for innovative interventions to identify men living with HIV, engage them in ART, and promote retention and adherence to achieve VLS. Strategies are needed to reach men who are at-risk and untested (in past 12-months) as well as men previously diagnosed but not adhering to treatment. Clinic service delivery was defined as being unfriendly to males, especially adolescent males [3–5].

Index partner testing strategies could be a high-yield way to identify men and link them to treatment. One randomized controlled study from Rosenberg et al in Malawi compared passive invitation to passive invitation plus contact tracing of male partners [6]. In that study, contact tracing was used to recruit men to get tested jointly with their partner thereby using CHCT as a mechanism for partner notification and mutual serostatus disclosure in the context of antenatal care [7].

Our study evaluated the impact of implementing a novel testing strategy, index partner/child testing, in which nurses and counselors were trained to track and trace partners and children of index patients living with HIV (recently diagnosed or on ART) implemented in public health hospitals and clinics in South Africa. We evaluated the impact of index partner/child testing on case finding and referral to ART.

## Methods:

To improve case finding, ART initiation, and HIV serostatus disclosure, we adapted the CDC/WHO couples HIV counselling and testing (CHCT) guidelines [8, 9] to focus on tracing and testing adult sex partners (15 years or older) and children (10–15 years old) of HIV-infected individuals in six high HIV prevalence Districts (Alfred Nzo, King Cetshwayo, Gert Sibande, Ugu, King Cethswayo, and Sedibeng Districts) in four Provinces (Eastern Cape, Gauteng, Kwa Zulu Natal, Mpumalanga). In our evaluation we excluded children less than 10 years old who were tested and reported through prevention of mother to child transmission intervention. During our pilot study, we trained nurses and lay counsellors working in HIV testing, antenatal care (ANC), ART and TB services in 56 large primary health care public facilities between March and December 2017. Clinics were primarily urban and peri-urban but we included some large, rural clinics. As we did not collect routine data from all sites prior to October 2017, this analysis includes data collected from October 2017 to June 2018when facilities had been trained and data collection was systematic. In addition to partner/child tracing and testing, our protocol included specific training on how to conduct CHCT for serodiscordant and concordant HIV-positive couples and their children, facilitate disclosure of serostatus results where one individual is HIV-infected, and how best to refer HIV-infected patients (or couples) to ART, or same-day ART services where available. The counselors were trained to screen for intimate partner violence or other potential social harms as a result of partner notification and HIV status disclosure. Anyone who reported IPV was referred to a social worker for counseling and legal support.

### Data collection:

During index patient post-HIV test counselling or ART adherence counselling (for patients already on ART) in ANC, TB and ANC services, counsellors and nurses asked index patients to refer their partner or children who did not know their HIV status or were not known to be on ART for testing. The provider would give the index patient three options for testing of their partner/child:

(1) the index patient invited their partner/child to test via a written invitation provided at the clinic which contained the index patient’s folder number or ID (which could lead to immediate testing if the partner/child was already in the clinic) but the partner/child returned alone for testing,
(2) the clinician called the partner to invite them for individual testing (without mentioning the index partner), or
(3) the index patient returned for testing with their partner or child, at which time they could test together or separately.

The above-mentioned options were not mutually exclusive, though the provider collected data only on the method that was effective at bringing the partner or child in for testing. Trained counsellors and nurses entered data in standardized government clinic HIV testing service logbooks after testing each participant and would note if the partner or child was referred by an index patient (self-reported). The partner/child was linked to the index patient if the counsellor or nurse received the written invitation with the index patient’s folder number or ID, or if the provider knew the index patient who was bringing their child or partner in for testing.

### Sub-analysis:

In a sub-analysis of a sub-sample of 10% of partner/children tested identified through a convenience sample of the total patients, we collected additional data on partner/child testing (where was testing provided, how was the partner/child was traced and if they were referred for ART). This data was collected on an electronic form as part of the patient electronic data from participants who tested following referral for partner/children testing. Participants were selected during a 2-month time period to compile additional information about the intervention and evaluate factors associated with HIV-positive diagnosis and referral to ART initiation.

### Quality control of data and data management:

Each facility had a supervisor who would observe the quality of the HIV counselling and testing services and review the HIV testing logbooks and electronic data for missingness or inconsistencies on a monthly basis. The supervisor would provide mentorship and re-training as necessary and clean the data with the provider to ensure that missing data was collected and inconsistencies corrected. The supervisors would review the HIV testing logbooks and compare to that in the index testing reports to ensure consistency.

### Data analysis:

We analysed routinely collected data from trained staff to report on findings between October 2017 and June 2018 including positivity by age and gender. Descriptive analysis of categorical variable using proportions summarized characteristics of partners and children of index patients who tested for HIV in the sub-sample of patients using Chi-square tests for differences in HIV positivity. We then modelled the outcome of HIV status of partner and child tested using univariate and multivariable logistic regression for partners and children in separate models adjusting for a priori confounders including gender and age of the partner/child tested.

### Human subjects considerations:

Program activities were implemented as part of routine primary healthcare and HIV testing and counselling. All data were retrieved from a de-identified retrospective analysis of patients’ electronic charts. Names, dates of birth, and ID numbers were removed from the dataset prior to analysis by the Department of Health staff. Participants provided informed consent to undergo HIV testing and counselling and for partner notification as part of the standard of care. University of California Los Angeles’s IRB provided exemption (UCLA IRB#19–000227).

### Role of the funding source:

This pilot program was funded by the United States Agency for International Development (USAID) under Cooperative Agreement AID-XXX-A-12–00016, managed by XXX. The donor had no involvement in the study design, data collection or analysis, interpretation of the results or writing of the report. The corresponding author had full access to all of the data in the study and had final responsibility for the decision to publish without involvement of the donor.

## Results:

**Figure 1:**
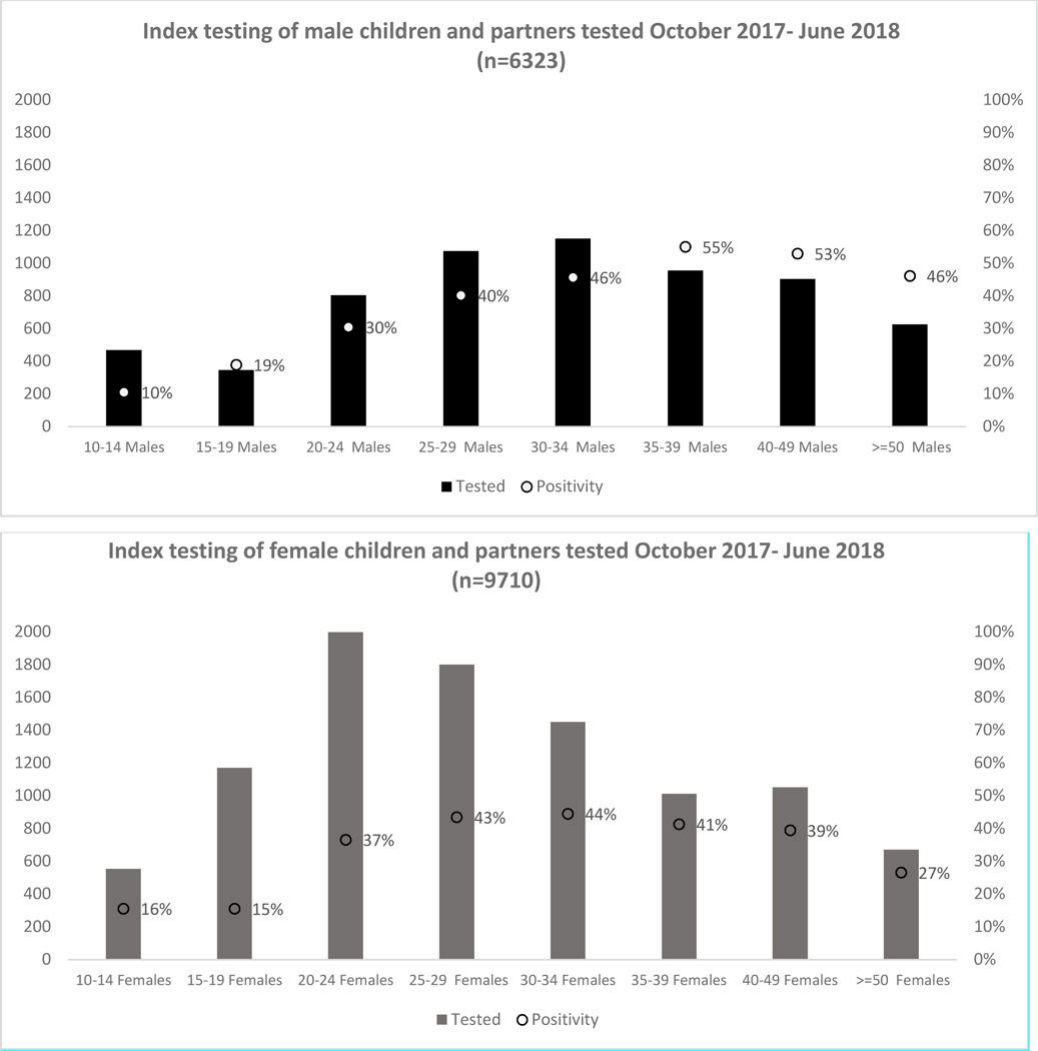
Partners and children of index patients tested by gender, age and positivity October 2017- June 2018 (n=16,033 partners and children tested)

During our pilot study, we trained 34 nurses and 52 lay counsellors working in HIV testing, ANC, TB services in 56 large primary health care public facilities between March and December 2017. Following training, providers tested 16,033 partners and children of index patients between October 2017 and June 2018. Most of those tested were female (61%; n=9710) and between 20–39 years old (39%, n=6263). Overall, 6.4% were 10–14 years old (n=1022), 9.5% were between 15 and 19 years old and 8% were 50 years or older. In total, 6038 of those tested were HIV-infected (38%; 95% CI=36%, 40%). In children 10–14, 13% were HIV-infected (95% CI=11%, 14%), 16% in females vs. 10% in males (p<0.05). In females, highest positivity was in 30–34 years old women (44%; 95% CI=42%−47%) followed by 25–29 years old (43%; 95% CI=41%−46%). In males, highest positivity was in 35–39-year-old men (55%; 95% CI=52%−58%) followed by 40–49 (53%; 95% CI=50%−56%) (Fig 1).

In the sub-set analysis of a convenience sample of 9.7% of patients (n=1554) in which we collected additional data, most patients were tested in public health clinics (98%) and 2% in public hospitals (n=34). Most partners and children were tested in voluntary counselling and testing services in the facility (65%), followed by the outpatient department provider-initiated testing (33%), antenatal care (1%), tuberculosis care (0.5%) and voluntary male medical circumcision (VMMC) (0.5%) (Table 1). Participants who tested after invitation from their partner (or with their partner) were identified in the HIV testing log from the counsellor, though they may have tested in TB or ANC services as part of the standard of care.

In terms of how the partner came into testing, 52% of index patients chose to ask their partners to get tested (using an invitation) but did not return to test with them, 24% were tested following phone tracing by the healthcare provider, 21% of index patients brought in their partner for individual or joint testing, and 3% was unknown. For children, 48% came in with their parent, 28% were tested following phone tracing by the healthcare provider, 21% of parents asked their children to come in for testing (and came in alone), and 3% was unknown. Overall 40% of index patients were counselled and tested together with their partner via CHTC. Almost all clients were referred for ART (97%), some receiving same-day initiation if they demonstrated they were ready to start ART (Table 1).

Odds of diagnosing an HIV-positive partner increased with their age (adjusted odds ratio [aOR] for increase in 5-year age category=1.21; 95% CI=1.04, 1.42), female gender (aOR=1.38; 95% CI=1.04, 1.82) and bringing the partner in for HIV testing vs. referring the partner through the provider or recommending testing to the partner (aOR=1.94, 95% CI=1.43, 2.63), adjusting for location of testing. Odds of diagnosing an HIV-positive child (under 15 years old) was highest with increased age (aOR=2.20, 95% CI=1.90, 2.55). In terms of location of testing, outpatient department provider-initiated testing had the highest odds of positivity (aOR=1.80, 95% CI=1.38, 2.34) versus voluntary counselling and testing adjusting for age and gender. Referral for ART initiation (97% were referred) did not differ by gender, age or location of testing service.

## Discussion:

**Figure 2.**
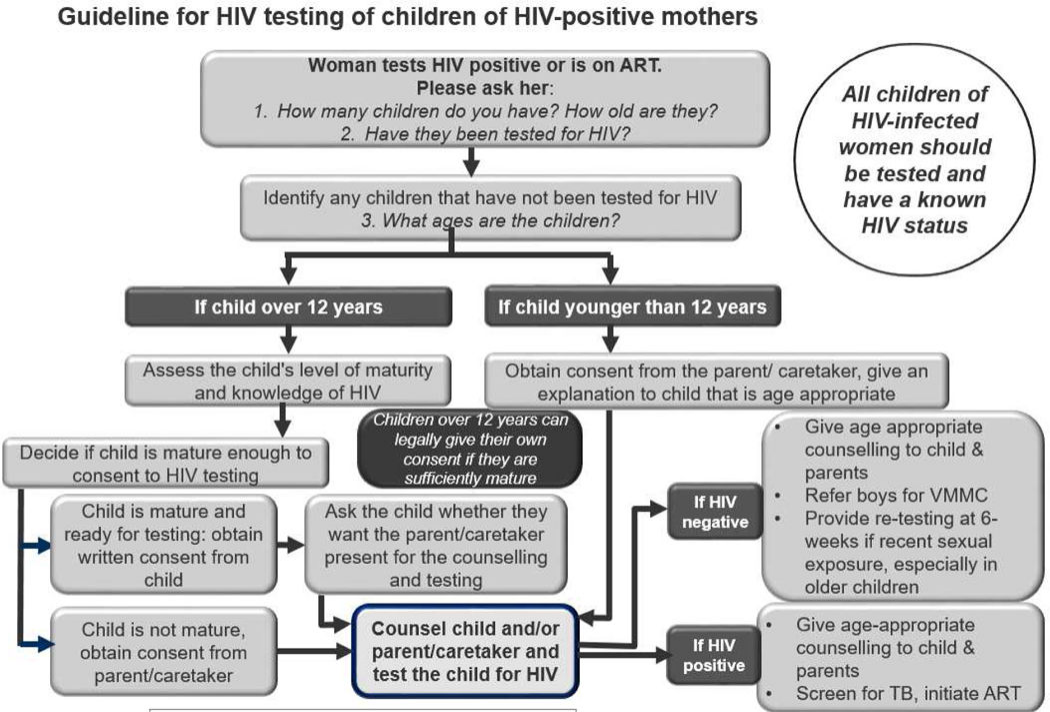
Guideline used for referral of children of HIV-positive mothers’ children to HIV testing services in South Africa

Testing of partners and children of index patients living with HIV, referred to as index testing, is an important high yield testing strategy that is essential to reach people living with HIV who may not know their status or know their status but are not yet on ART, especially men who remain disproportionately underdiagnosed in South Africa [1]. The positivity in index testing was very high; almost half of partners of index cases were HIV-infected. Further, children 10–14 years old had a very high positivity of 13%. In South Africa, children 12 and over can consent for HIV testing on their own if the provider deems them to be sufficiently mature [18], see Figure 2 for HIV testing guidelines used for children of HIV-infected mothers. Most index testing occurred in voluntary counselling and testing services, yet testing of index partners in the outpatient departments in public health facilities yielded more HIV-infected partners. The most successful mode of invitation was the index patient inviting their partner/child to return for testing (or they were recruited/tested during an index partner clinical visit which they were attending). Integrating index partner/child testing into public health facilities was feasible and effective at increasing the positivity of HIV testing services from 8% prior to the pilot to 41% in sex partners of index patients.

Our pilot reached 2605 HIV-positive males (41% of 6323 males tested) in 9 months and referred almost all men to initiate ART. Index partner testing in the outpatient departments in public health facilities yielded more HIV-infected adult male partners compared to other services including VCT. In Tanzania, successful partner referral was 2.2 times more likely among male compared to female index clients [11]. As in Tanzania, women may need additional support to overcome challenges in the partner notification process, especially in a context of high interpersonal violence in South Africa [15]. Several studies have integrated male partner and couples HIV testing into antenatal care (ANC) yielding mixed results [16, 17]. Similarly, we had very low uptake of male partner testing in ANC. HIV self-testing may be another option to improve HIV testing uptake in male partners of HIV-infected women [14].

Almost half of index patients chose to invite their partners to get tested themselves (similar to studies in Malawi where index-partner initiated strategies were preferred over provider-initiated contact [7]), and one-third of index cases came with their partners for individual or joint testing. However the recent systematic review of partner notification found that provider-initiated strategies were most effective in terms of yield and linkage [2]. This has implications for the role out of index testing in that it may be less expensive and simpler to empower index patients to disclose their HIV status and refer their partner to come in for testing. However female index cases and those with limited power may be left out if they are unable to discuss HIV with their partner(s) and/or children for fear of reprisal, abuse or abandonment. We advocate for providing both provider and index patient led strategies to improve disclosure and index testing, including couples testing and counselling [6].

PEPFAR released a toolkit on index and partner notification in April 2018 which includes job aids, talking points and tools for documenting and monitoring partner notification services [13]. However, the toolkit does not discuss the potential for couples counseling and testing to conduct index partner testing, which our study found to be a popular option. We found that couples’ counselling was also popular and described by participants to receive accurate information and reduce potential conflict between partners. The logistics of how couples counseling occurs when one partner already knows their serostatus (and may be on ART) is challenging and may require re-testing of the HIV-infected partner if they have not yet disclosed their status. Future studies should explore how best to invite, jointly test, and link to care the partners of index patients living with HIV [3].

Limitations of our study include that we collected programmatic data with counsellors and nurses. We did not collect data on index patients who declined to invite their partners/children for testing or were not successful in partner tracing and testing We did not collect data on ART initiation and only report on referrals to ART. Further, we did not confirm if the partner or child had been tested before and already knew their HIV status. This may have biased our results to overestimate the true positivity of those tested. Further there is potential for bias in the results and positivity due to convenience sampling.

## Conclusion:

Integrating index testing into HIV care was feasible and we identified a very high yield when testing partners and children of index patients. Index testing should be provided to all people diagnosed with HIV, whether or not they have initiated ART, to provide opportunities to diagnose those at highest risk of being infected with HIV who may still be unaware of their status. There is an urgent need for policies and training to be rolled out to implement index partner and children testing at scale. Additional implementation research is needed to evaluate which implementation methods (for example, comparing the effectiveness and costs for clinic-based, community-based, and self-testing strategies) are most effective and cost-effective at reaching partners and their children of index patients and linking those diagnosed to treatment.

## Figures and Tables

**Table 1.** Characteristics of partners and children of HIV-positive patients (index patients) testing for HIV by HIV status of partner/child tested (n=1554 patients with additional data collected)

	Overall (n=1554) *	HIV-negative (n=889)**	HIV-positive (n=665)**	p-value
**Who was traced: partner or child?**				
Partner	911 (59%)	331 (36%)	580 (64%)	<.0001
Child	643 (41%)	558 (87%)	85 (13%)	
				
**How was client traced (of partners traced & tested; n=911)?**				
Health care provider contacted partner	220 (24%)	99 (45%)	121 (55%)	<.0001
Patient came in for testing with partner	193 (21%)	80 (42%)	113 (58%)	
Patient told partner to come in for testing	473 (52%)	141 (30%)	332 (70%)	
Don’t know/ unsure	25 (3%)	11 (44%)	14 (56%)	
				
**How was client traced (of children traced & tested; n=643)?**				
Health care provider followed up	182 (28%)	174 (96%)	8 (4%)	<.0001
Child came in for testing with parent	309 (48%)	270 (87%)	39 (13%)	
Patient told child to come in for testing	132 (21%)	97 (73%)	35 (27%)	
Don’t know/ unsure	20 (3%)	17 (85%)	3 (15%)	
				
Partner tested with index partner (couple testing)***				
Yes	360 (40%)	129 (36%)	231 (64%)	0.800
No	551 (60%)	202 (37%)	349 (63%)	
				
**Where was partner or child tested? (n=1521)**				
Antenatal care	22 (1%)	18 (82%)	4 (18%)	0.0001
Outpatient care	502 (33%)	241 (48%)	261 (52%)	
Tuberculosis services	9 (0.5%)	3 (38%)	5 (63%)	
Voluntary counseling and testing services	982 (65%)	605 (62%)	377 (38%)	
Voluntary medical male circumcision	6 (0.5%)	4 (0.5%)	2 (0.3%)	
				
**Referred for ART start**				
Yes	646 (97%)	N/A	646 (97%)	N/A
No	19 (3%)	N/A	19 (3%)	
				

* column %

** row %

*** removed children testing
